# Supplementary material for: Proper Selection of In Vitro Cell Model Affects the Characterization of the Neutralizing Antibody Response against SARS-CoV-2
Source: Viruses. 2022 Jun 7;14(6):1232. doi: 10.3390/v14061232 (PMC9230092; doi:10.3390/v14061232)
Supplement: Supplementary file 1 [file viruses-14-01232-s001.zip › viruses-1733335-supplementary.pdf]

Supplementary material

Table S1. Demographic information on the HCP sera used in the study.

Serum #	Gender	Age	Underlying health problem
2	M	52	Hyperglycemia not in treatment
5	M	72	BPH
17	M	84	Prostatectomy, bilateral inguinal hernioplasty, arterial hypertension, phacoemulsification with bilateral IOL graft, subarachnoid hemorrhage secondary to ruptured aneurysm, acute cholecystitis
26	M	86	Bronchiectasis, former smoker, surgery for lumbar disc herniation, TURP for BPH
32	M	58	None
25	M	51	Diabetes mellitus
29	F	60	Diabetes mellitus, arterial hypertension, hearing loss
37	M	59	Hypertension

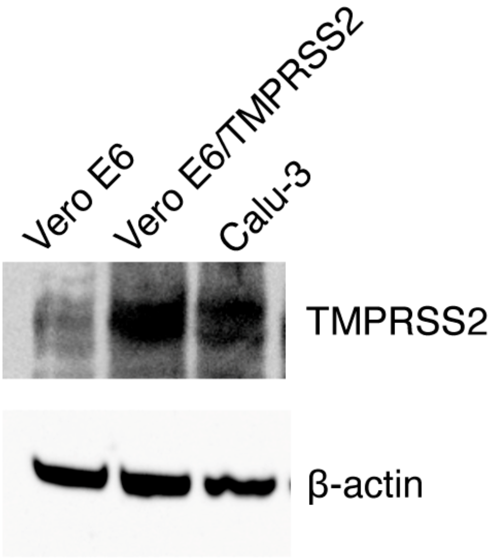

Figure S1. Expression level of TMPRSS2 protein in the different cell lines. Lysate cells were analysed through Western Blot to assess the expression of TMPRSS2 protein. β-actin was used as housekeeping control.

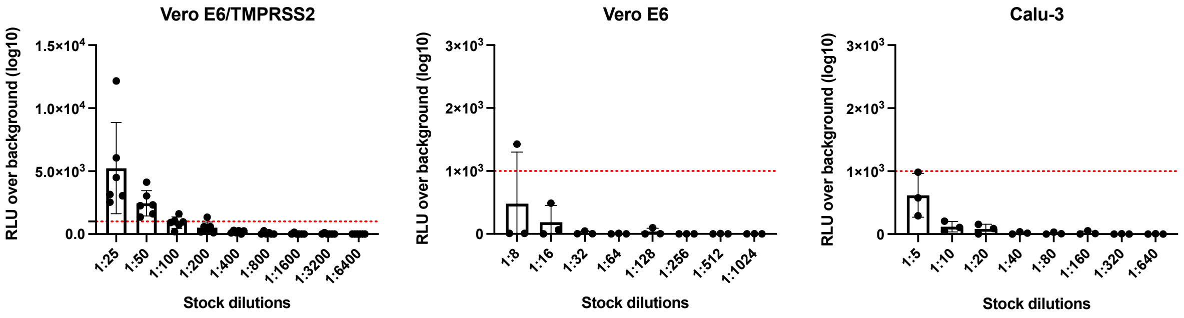

Figure S2. Pseudovirus titration in the selected cell lines. Each condition was tested six times, and data were normalized over the background. Values were reported as mean values ± SD, cut-off value 10^3 RLU over background (red dotted line).

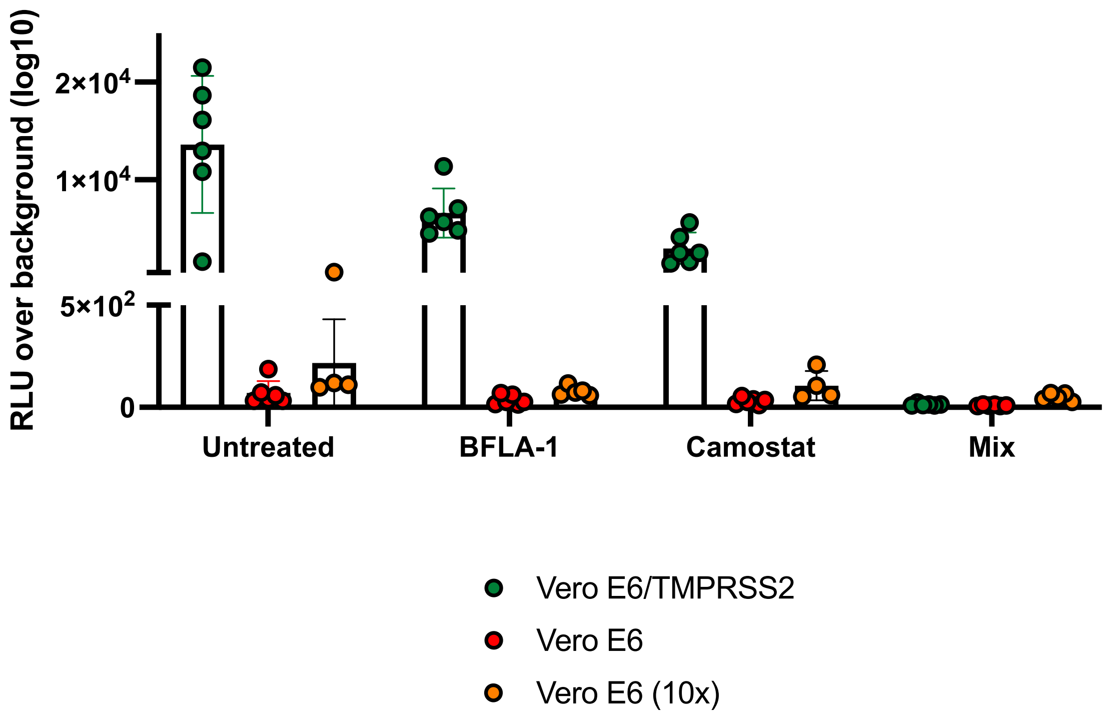

Figure S3. Inhibition of pseudovirus entry. BFLA-1 and camostat mesylate were used, alone and in combination, to hamper the entry of pseudotyped virus particles into wild type and engineered Vero E6 cells. Vero E6 (red dots), Vero E6/TMPRSS2 (green dots), Vero E6 infected with 10 times as much pseudovirus particles (orange dots). Each condition was tested six times, data were normalized over the background. The infection inhibition percentages were reported as mean values ± SD.

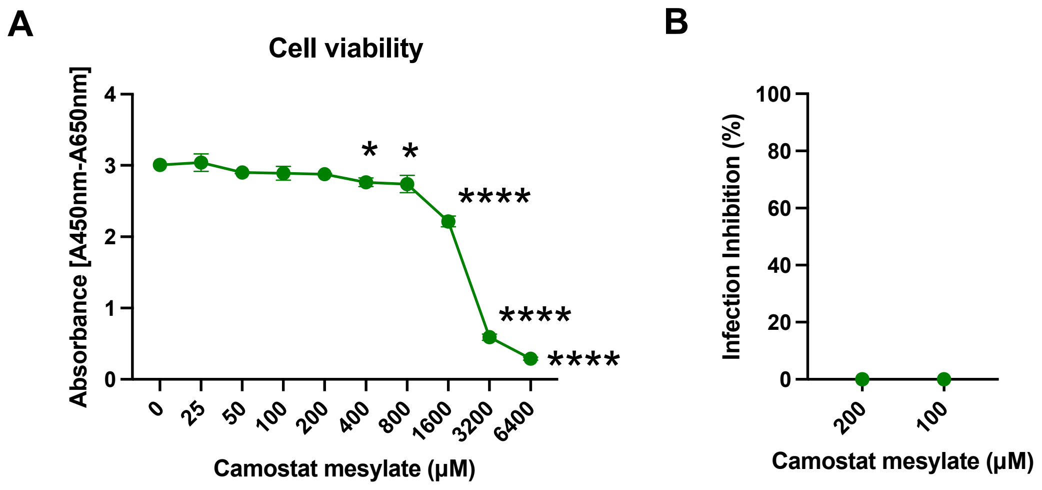

Figure S4. Effect of camostat mesylate. A) Different concentrations (25-6400 µM) of camostat mesylate were tested to assess their toxicity on Vero E6/TMPRSS2 cells. Absorbance was reported as mean values ± SD, * $p<0.05$, ****$p<0.0001$. B) The two higher non-toxic concentrations were tested against virus infection. The infection inhibition percentages are reported as mean values ± SD.
